# The protective effects of rapamycin on cell autophagy in the renal tissues of rats with diabetic nephropathy via mTOR-S6K1-LC3II signaling pathway

**DOI:** 10.1080/0886022X.2018.1489287

**Published:** 2018-09-11

**Authors:** Lei Liu, Lijuan Yang, Baochao Chang, Jiqiang Zhang, Yaling Guo, Xiangdong Yang

**Affiliations:** a Department of Nephrology, Shandong University Qilu Hospital, Jinan, P.R. China;; b Department of Physiology, Bengbu Medical College, Bengbu, P.R. China;; c Department of Nephrology, The First Affiliated Hospital of Bengbu Medical College, Bengbu, P.R. China

**Keywords:** Diabetic nephropathy, podocyte, autophagy, mTOR, rapamycin

## Abstract

**Background:** Previous studies have shown that podocyte autophagy is an important trigger for proteinuria and glomerulosclerosis. The mammalian rapamycin target protein (mTOR) occupies a pivotal position in the autophagy pathway. In this study, we planned to clarify the mechanism of mTOR regulation of podocyte autophagy and the effect of rapamycin (RAPA).

**Methods:** All rats were randomly divided into normal control group (*n* = 8), DN group (*n* = 8), and RAPA group (*n* = 8). Blood and urine samples were collected at the 4th, 8th, and 12th weeks of the experiment. The serum creatinine (Scr), urine volume levels, and the 24 h urine protein (UP) levels were examined. The nephrin, podocin, mTOR, ribosomal S6 kinase 1 (S6K1), and autophagy marker light chain 3 (LC3II) expression levels were evaluated by immunohistochemistry, quantitative PCR, and immunoblotting.

**Results:** The urine volume, 24 h UP, and Scr of the DN and RAPA groups increased significantly compared with the NC group (*p* < .05). Nephrin and podocin expression was decreased in the kidney tissues of the DN and RAPA groups compared with the NC group (*p* < .05). The expression levels of mTOR and S6K1 increased and LC3II expression decreased in the renal tissues of the DN and RAPA groups compared with the NC group (*p* < .05). After RAPA treatment, all the above indexes were improved compared with the DN group (*p* < .05), but were significantly abnormal compared with the NC group (*p* < .05).

**Conclusion:** The proteinuria and kidney function had improved after RAPA treatment. These results confirmed that RAPA specifically binds to mTOR kinase, and inhibits mTOR activity, thereby regulating the pathological autophagic process.

## Introduction

Diabetic nephropathy (DN) is a serious complication of diabetes mellitus (DM). The clinical manifestations of DN are proteinuria, renal dysfunction, and finally progression to end-stage renal disease. Podocytes, which are the key structures of the glomerular filtration barrier, play an important role in preventing leakage of plasma proteins. Studies have shown that the damage and loss of podocytes seriously damages the integrity of the glomerular filtration barrier, promote glomerular sclerosis, changes the phenotype and function of podocytes, and accelerates the progression of diabetic nephropathy [1]. As an inherent physiological function of eukaryotic cells, autophagy is an important mode of cellular degradation and is essential for maintaining the intracellular environment and for cell survival. Under normal conditions, podocytes exhibit high levels of autophagic activity, but cell autophagy can change under pathological conditions. mTOR occupies a pivotal position in the autophagy pathway; for instance, mTOR activation inhibits autophagosome formation, and many factors affect autophagy when mTOR activity is altered [2]. mTOR activation regulates the expression of LC3II, ribosomal S6 kinase 1 (S6K1) and other proteins, leading to podocyte injury [3]. Rapamycin (RAPA) can specifically bind to the mTOR kinase and inhibit mTOR activity, thereby regulating the pathological autophagic process [4]. In this study, we investigated the pathogenesis of diabetic nephropathy from the perspective of autophagy and used RAPA to intervene rats. In addition to proving that RAPA can inhibit mTOR to improve autophagy, we wanted to clarify the autophagy pathway mOTR-S6K1-LC3II. This might provide a new therapeutic direction for the clinical treatment of diabetic nephropathy.

## Material and methods

### Major reagents and sources

Streptozotocin (STZ) was purchased from Sigma (No. 101764603); RAPA was purchased from Shanghai Baoman Biotechnology Co., Ltd. (No. 53123–88-9); Anti-nephrin and anti-podocin primary antibodies were purchased from Beijing Boao Sen (bs-0513R and bs-6597R). RNA extract was purchased from Servicebio (Cat. No. G3013); The RevertAid First Strand cDNA Synthesis Kit was purchased from Thermo (No.K1622). The FastStart Universal SYBR Green Master (Rox) was purchased from Roche (No. 04913914001). The BCA Protein Quantitative Detection Kit was purchased from Servicebio (NO.2026). The Protein Marker was purchased from Thermo (NO.26616). The horseradish peroxide labeled goat anti-rat antibodies were purchased from Servicebio Co., Ltd. (GB23301 and GB23302).

### Animals and experimental design

Experimental male Sprague-Dawley rats were provided by Bengbu Medical College Animal Center (Bengbu, Anhui, China). All rats were randomly divided into normal control group (*n* = 8), a DN group (*n* = 8), and a RAPA group (*n* = 8). The rats were kept in a standard laboratory; with 50–65% humidity 20–24 °C, with a 12 h light/dark cycle. The rats were provided free access to food and water. The DN and RAPA groups were administered 60 mg/kg of STZ via intraperitoneal injection. For 3 d after the injection, the blood glucose levels of the rats were evaluated. Blood glucose levels higher than 16.7 mmol/L were required [5]. The RAPA group was intraperitioneally injected with 2.0 mg/kg·d of RAPA, and the NC and DN groups were intraperitioneally injected with citrate buffer. This study was approved by the Bengbu Medical College Ethics Committee (Batch number: 2016094).

### Blood and urine collection and examination

Blood and urine samples were collected at the 4th, 8th and 12th weeks of the experiment. The serum creatinine (Scr) and urine volume levels were measured, and the 24 h urine protein (UP) levels were examined. The rats were sacrificed at 12 weeks after anesthesia, and the kidneys were collected and preserved. Immunohistochemistry, quantitative PCR, and immunoblotting were performed.

### Immunohistochemistry analysis

Dewaxed the paraffin sections with xylene and ethanol then placed the sections in citric acid buffer for antigen repair. After blocked the endogenous peroxidase by hydrogen peroxide, sealed the sections with serum, then anti-nephrin and anti-podocin primary antibody had been added. Before hematoxylin staining, the sections had been colored by the DAB liquid. At last, the sections were observed under light microscope after dehydrated with alcohol and sealed with neutral balsam.

### RNA extraction and fluorescent quantitative PCR analysis

RNA extraction was performed in homogenate tubes containing 1 mL of the TRIzol reagent and 100 mg of tissue. The mixture was fully ground until no visible tissue remained, and then centrifuged at 12,000 rpm. Then, chloroform was added, and the mixture was centrifuged again at 12,000 rpm. After centrifugation, isopropyl alcohol was added to the mixture at 0.8 times the volume and the RNA was dissolved in RNase-free water. Excessively high RNA concentrations were diluted to a final concentration of 200 ng/μl. A NanoDrop 2000 was used to measure the RNA concentration. Fluorescent quantitative PCR was performed with oligo-(dT)-18, reaction buffer, dNTP Mix, the RiboLock RNAase inhibitor, and the RevertAid Moloney murine leukemia virus reverse transcriptase. The PCR reaction mixture was incubated at 42 °C for 60 min; and then at 70 °C for 5 min to inactivate the reverse transcriptase. A reaction system was prepared for PCR amplification, including primers, the reverse transcription product, and ddH2O, and a melting curve was generated.

### Primer information

The primer sequences used in the present study were synthesized by Wuhan servicebio technology CO., LTD. Sequences were as follows: (1) podocin, Forward, 5′-GC CTCCCTTCTT CTAAGCAGTCTA-3′; Reverse, 5′- TCAGTTCTCTCCACTTTGATGCC-3′; (2) nephrin, Forward, 5′-GACA CGAGAAGCTCCACGGTTA-3′; Reverse, 5′-GTCGTAGATTCCCCTCGGATC-3′; (3) mTOR, Forward, 5′-TTGCCAACTACCTTCGGAACC-3′; Reverse, 5′-TCA CGGAGAACGA GGACA GC-3′; (4) LC3II, Forward, 5′-AGAGCGATACAAGGGTGAGAAG-3′; Reverse, 5′-AGAAGGCTTGGTTAGCATTGAG-3′; and (5) S6K1, Forward, 5′-ACAGCCCC GATGACTCAAC TCTC-3′; Reverse, 5′-CGTGGGCTACCAATAAATCTTCG-3′.

### Western blotting analysis

The renal tissue was cut into pieces, and phenylmethylsulfonyl fluoride was added to produce a tissue homogenate. The homogenized tissue was vortexed at the highest available speed for 5 s. Then, the cell pellet was completely suspended and dispersed, the cytoplasmic protein extraction reagent was added, and the mixture was again vortexed at the highest speed for 5 s. After centrifugation for 5 min, the supernatant was transferred to a pre-cooled plastic tube. Western blotting analysis was performed with electrochemiluminescence reagent A (ECLA), ECLB, polyvinylidene difluoride membrane, 5% skimmed milk, a primary antibody, and a secondary antibody mixed in a centrifuge tube. The membrane was exposed scanned, archived, and colored using Photoshop. Alpha software was used to process the images and assess the optical density values.

### Statistical analyses

All analyses were performed using SPSS v. 20.0. Quantitative data were expressed as the mean ± SEM. An independent-samples *t*-test or ANOVA was applied for the statistical analysis. Pair-wise comparisons were performed using *t*-tests. Significance was set at *p* < .05.

## Results

### RAPA alleviated proteinuria and histological damage in the kidneys of DM rats with DN

The 24 h UP and Scr levels were measured in the NC, DN, and RAPA treatment groups in the first (termed week 0), 4th, 8th, and 12th weeks. Compared to the NC rats, the DN rats had a markedly higher 24 h UP and Scr level at 8th week that maintained for the entire study period (12 weeks). The urine volume of the DN and RAPA groups increased significantly compared with the NC group, which related to the effect of the hyperglycemia. At the end of the study, we came to conclusion that the glomerular filtration rate of the DN and RAPA group rats had decreased significantly, and the proteinuria and the glomerular filtration rate in the RAPA group was alleviated compared with the DN group, which may relate to the effect of RAPA (Tables 1–3).

### Podocyte damage in DN rats

**Figure 1.**
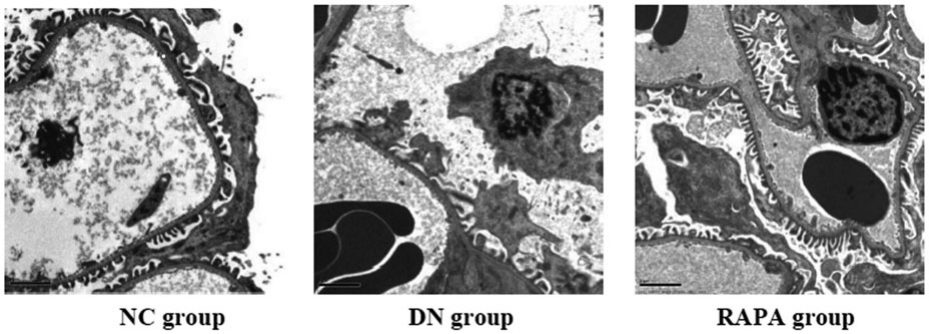
The podocyte pathology changed in the three groups under the electron microscope.

**Figure 2.**
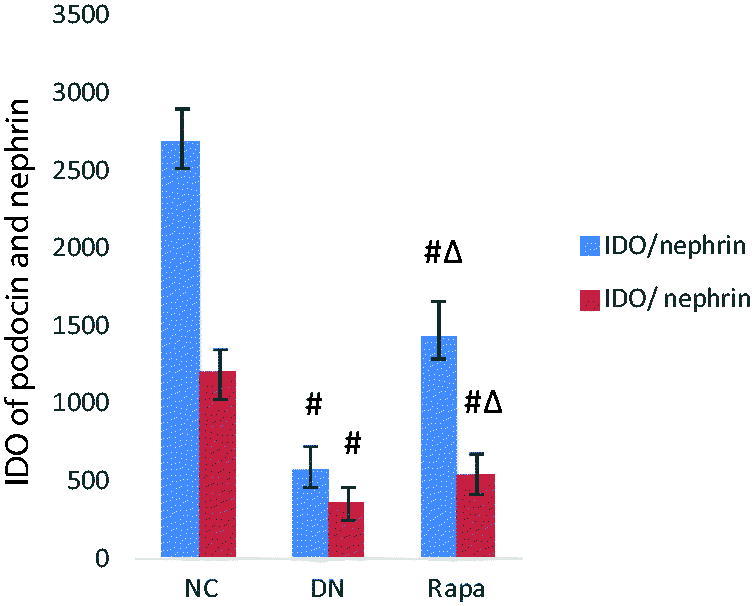
The podocin and nephrin expression in the kidney tissues of the three groups detected by immunohistochemistry.

**Figure 3.**
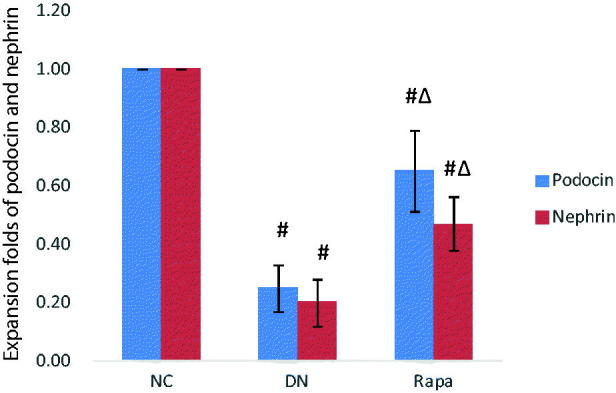
The podocin and nephrin expression in the kidney tissues of the three groups detected by quantitative PCR.

Electron microscope revealed that the podocyte pathology changed in the three groups. Compared with the NC group, the DN group had ablated podocyte foot process structures and reduced numbers of podocyte foot processes. In the RAPA group, less ablation of podocyte foot processes was observed in the DN group than that in the NC group (Figure 1). Podocin and nephrin which are podocyte marker proteins, reflect podocyte damage. The expression levels of these two proteins and their associated genes were assessed by immunohistochemistry and quantitative PCR in the kidney tissues of rats from the three groups. The PCR of podocin and nephrin expression in the kidney tissues are improved significantly in the RAPA group compared with the DN group rather than the NC group (*p* < .05) (Figure 2). PCR showed significant differences in podocin and nephrin expression in the kidney tissues of rats from the three groups, with the expression levels significantly lower in the DN group *(p* < .05) and significantly greater in the RAPA group (*p* < .05) than those in the NC group (Figure 3).

### Changes in autophagy and the effects of RAPA on autophagy

**Figure 4.**
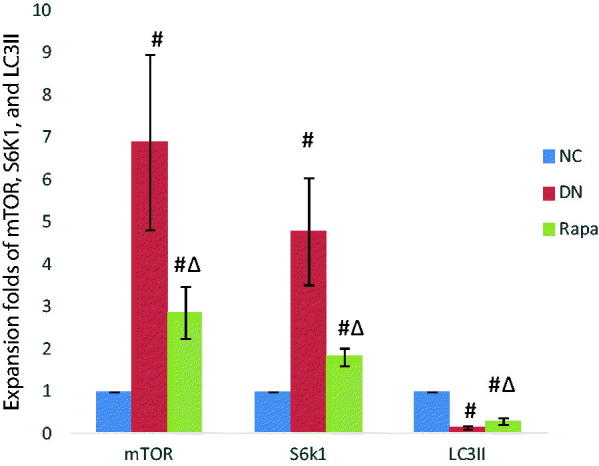
The mTOR, S6K1 and LC3II expression in the kidney tissues of the three groups detected by quantitative PCR.

**Figure 5.**
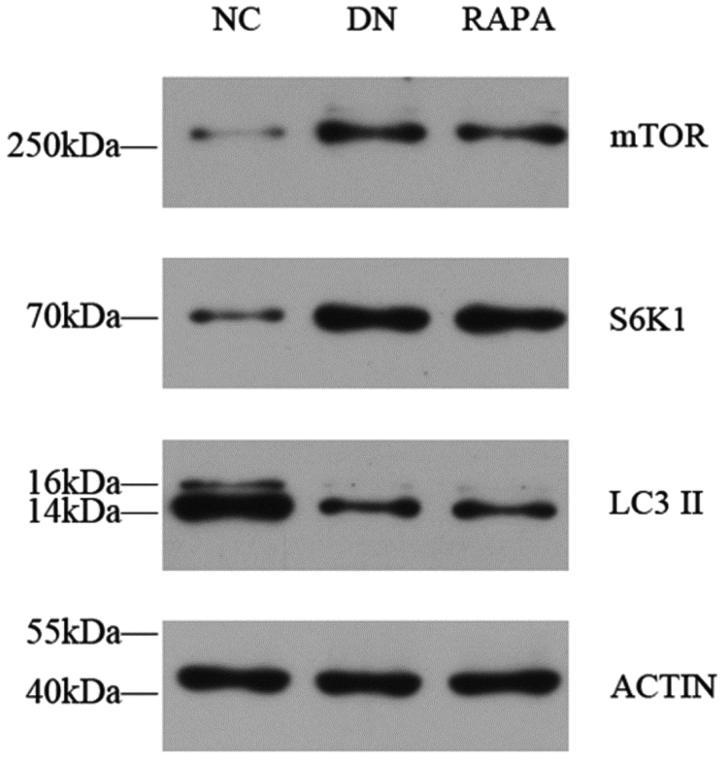
The mTOR, S6K1 and LC3II expression in the kidney tissues of the three groups detected by western blotting.

Podocytes are impaired in diabetic individuals and autophagy levels are affected by a variety of factors. mTOR protein activation affects downstream protein expression pathways, directly resulting in a decrease in the number of autophagosomes. The immunofluorescence, PCR, and western blotting results showed that the mTOR protein expression was significantly higher in the DN group than that in NC group (*p* < .05), whereas expression in the RAPA group was improved compared with expression in the DN group (*p* < .05) (Figure 4). LC3II expression showed the opposite profile with the LC3II levels lower in kidney tissues in the DN group than those in the NC group (*P* < .05). The LC3II changes were not directly affected by mTOR, which might use a separate pathway. S6k1 protein expression was significantly higher than in the DN group than that in the NC group (*p* < .05) and was increased in the RAPA group (*p* < .05), suggesting that mTOR might be affected by the S6k1 protein, with downstream effects on autophagy and LC3II expression (Figure 5, Table 4).

**Table 1.** Urine volume analysis results in the three groups.

Group	Number	Week 0	Week 4	Week 8	Week 12
NC	8	0.0029 ± 0.0008	0.0031 ± 0.0001^a^	0.0109 ± 0.0044^a^	0.0083 ± 0.0028^a^
DN	8	0.0028 ± 0.0012	0.1216 ± 0.0548^b^	0.1755 ± 0.0430^b^	0.2206 ± 0.0479^b^
RAPA	8	0.0031 ± 0.0013	0.1263 ± 0.0302^b^	0.1643 ± 0.0401^b^	0.1706 ± 0.0221^c^
*F*		0.149	29.891	58.469	106.205
*P*		0.863	0	0	0

Note: The symptoms of polyuria can be observed from the beginning of the week 4 in the DN and RAPA groups rats, and this conform to the performance of diabetes (*P* < 0.05). At the end of the week 12, the urine volume of the RAPA group rats has reduced compared with the DN group (*P* < 0.05), which may be related to the disease under control.

**Table 2.** Urine protein analysis results in the three groups.

Group	Number	Week 0	Week 4	Week 8	Week 12
NC	8	0.0031 ± 0.0013	0.0037 ± 0.0012^a^	0.0052 ± 0.0025^a^	0.0056 ± 0.0020^a^
DN	8	0.0033 ± 0.0011	0.1176 ± 0.0558^b^	0.1696 ± 0.0840^b^	0.1939 ± 0.0678^b^
RAPA	8	0.0038 ± 0.0008	0.1110 ± 0.0278^b^	0.1199 ± 0.0350^b^	0.1250 ± 0.0313^c^
*F*		0.881	25.237	20.578	38.971
*P*		0.429	0	0	0

Note: Proteinuria is a typical clinical manifestation of diabetic nephropathy. It could be observed that proteinuria increased significantly in the DN group from 4th week compared with the results in the NC group (*P* < 0.05). With the development of the disease, the results of urinary protein increased significantly in the 12th week compared with the 4th week (*P* < 0.05), which was consistent with the characteristics of the disease. Compared with the NC group, urinary protein quantification increased significantly in the RAPA group from 4th week (*P* < 0.05), but decreased significantly compared with the DN group at the end of the 12th week (*P* < 0.05), which was related to the effect of RAPA.

**Table 3.** Blood creatinine analysis results in the three groups.

Group	Number	Week 0	Week 4	Week 8	Week 12
NC	8	43.7826 ± 10.2562	47.1250 ± 9.7605^a^	47.7500 ± 8.3109^a^	51.2500 ± 5.9702^a^
DN	8	41.2367 ± 8.1289	55.3750 ± 13.2874^b^	80.8750 ± 17.1167^b^	119.2500 ± 28.6394^b^
RAPA	8	44.2918 ± 7.6721	51.7500 ± 11.2285^b^	73.6250 ± 9.5310^b^	100.1250 ± 10.7230^c^
*F*		0.279	1.094	16.069	30.401
*P*		0.759	0.353	0	0

Note: The persistent proteinuria can cause glomerulosclerosis, which decrease glomerular filtration rate and increased blood creatinine. In this study, we found that the blood creatinine of the DN and RAPA groups increased gradually from the 4th week compared with the NC group (*P* < 0.05). At the end of the 12 week, the blood creatinine of the RAPA group decreased compared with that of the DN group, which may be related to the effect of RAPA (*P* < 0.05).

**Table 4.** mTOR, S6K1, LC3 II amplification factor analysis detected by western blot.

Group	Number	mTOR	S6k1	LC3II	ACTION
NC	8	0.21 ± 0.037^a^	0.36 ± 0.054^a^	1.46 ± 0.087^a^	1
DN	8	0.73 ± 0.045^b^	0.97 ± 0.087^b^	0.57 ± 0.074^b^	1
RAPA	8	0.63 ± 0.055^c^	0.82 ± 0.043^c^	0.65 ± 0.077^b^	1
**F**			200.985	295.555	
**P**			0	0	

Note: AlphaEase FC software was used to detect gray-scale value of sreaps to observe the expression of mTOR, S6K1 and LC3 II.

## Discussion

As one of the key structures of the glomerular filtration membrane, podocytes play an important role as a barrier and can effectively prevent leakage of plasma proteins. Many studies have suggested that a reduce number of podocytes, and structural and functional changes are the bases for DN progression and glomerulosclerosis. Diabetes is a common risk factor, leading to podocyte damage and the accumulation of proteins and organelles, which result in cytotoxicity and can induce apoptosis or necrosis if not expediently removed. In turn, these conditions cause irreversible podocyte injury and dysfunction, which decrease the number of podocyte [6]. Podocyte damage and loss seriously damage the integrity of the glomerular filtration barrier, leading to proteinuria and podocyte injury and promoting glomerular sclerosis, which accelerate the progression of diabetic nephropathy [7]. In our study, we found significant differences in the morphological, structural, and functional aspects of podocytes in the kidney tissues of rats with DN compared with those in the NC group. Nephrin and podocin which are podocyte-specific marker proteins, with ion channel and signal transduction functions, play important roles in podocyte morphology, tissue structure, and functional regulation of the foot septal membrane [8]. Podocyte damage occurs, and the expression levels of these two markers of protein are altered in diabetic individuals. The immunohistochemistry and quantitative PCR analyses demonstrated that the rats in the DN group exhibited significantly decreased nephrin and podocin protein expression levels than the NC group confirming that diabetes induced kidney cells damage.

Autophagy is a cellular self-protection mechanism that occurs when aged or damaged cells accumulate damaged organelles and macromolecules. The autophagy levels decreased in individuals with DN, leading to the intracellular accumulation of organelles and macromolecules that could not be cleared, and thus podocyte injury [9–10], it was worth to be study. The decrease in autophagy in the DN group compared with the NC group was the core focus of our study. Autophagy is regulated by a variety of signaling channels. mTOR occupies a pivotal position in autophagy signaling pathways and is the initial focus of this study. mTOR is an important serine/threonine protein kinase that plays a negative regulatory role in autophagy. mTOR activation can inhibit autophagosome formation, and its upstream regulatory factors include growth factors, insulin levels, nutrition, metabolic conditions, and mechanical changes. Changes in these factors are widespread in diabetic individuals and have been confirmed in many studies [11]. Activated mTOR regulates mRNA translation, ribosomal biosynthesis, protein translation initiation, and other important physiological functions in podocytes in diabetic individuals by phosphorylating the ribosomal S6 kinase 1 (40S ribosomal 6 kinase 1, S6K1) and the eukaryotic initiation factor 4E binding protein 1 (4EBP1) [12,13], which reduce autophagy. LC3-II, which is a marker of mammalian autophagosomal membrane proteins, can reflect changes in autophagy [14,15]. In our study, we evaluated mTOR, S6K1, and LC3II protein expression in rat kidneys and confirmed the important role of the mTOR-S6K1-LC3II pathway in DN renal injury. The development of DN may be related to the damage and destruction of podocytes induced by autophagy, this change in autophagy is associated with mTOR activity in the podocyte. So, maintenance of autophagy through inhibition of podocyte mTOR activation may be an important therapeutic target for DN [16].

RAPA is a commonly used drug to prevent clinical rejection after organ transplantation. RAPA binds to the mTOR kinase to inhibit mTOR activity and regulate autophagy [17]. RAPA has been found to increase the autophagic activity of kidney podocytes in diabetic mice and inhibit podocyte apoptosis [18]. Our results showed that the mTOR and S6K1 protein expression was decreased in the kidney tissues of rats after RAPA treatment. The results show that RAPA decreased the urinary protein levels in DN rats and mitigated podocyte injury via a mechanism that might be related to the regulation of mTOR expression by RAPA, which affected podocyte autophagy through a pathway related to the mTOR-S6K1-LC3II signaling pathway.

## Conclusion

These results confirmed that the autophagy was do the vital function in the pathogenesis of DN. RAPA specifically binds to mTOR kinase and inhibits mTOR activity, thereby regulating the pathological autophagic process.
